# Tumors Resulting from Septic Pulp of Teeth

**Published:** 1898-02

**Authors:** G. Lenox Curtis

**Affiliations:** New York.


ARTICLE VI.

### TUMORS RESULTING FROM SEPTIC PULP OF TEETH.

BY G. LENOX CURTIS, M. D., NEW YORK.

The object of this paper is to illustrate several of the most prevalent tumors of the jaw, evolved by septic material discharged from the decomposing pulp of the teeth. It is my intention to speak of them from a practical standpoint, and the desire is to inform those interested how to treat and prevent their recurrence.

Experience leads me to believe that the origin of fully ninety per cent. of all tumors of the jaw, face and neck can be traced to diseases of the teeth, and in many cases to the septic pulp. The most common of these tumors is the alveolar abscess, the contents varying from mere gas to to thick pus. The treatment has been described so often that I shall not here repeat it, further than to say—remove the cause and prevent recurrence. This can be done by cleansing the pulp-canal, filling it perfectly, dissecting away the sac, roughening the surface of the bone sufficiently, and promoting healthy granulation to fill the cavity. Another treatment is by extracting the tooth; this course, which is so often considered sufficient, does not always effect a cure. Whenever a decomposed pulp exists a cavity in the jaw will be found, at or near the apex of the root. The exception, I believe, is only when the peridental membrane has not been inflated so as to produce an abscess sac, but has immediately succumbed to the septic influence of the pulp, the gas passing through the canals of the bone to some remote part, and there forming the tumors. This may be so gradual that the attention of the patient has not been called to it, except by the soreness and swelling that might result from the primary attack, and is recalled by the patient only when questioned about it. These cysts may be simple or multiple, and I have seen as many as three in one-half of the upper jaw.

Whenever the pulp of a tooth is found to be devitalized, and is not removed and filled as suggested, you can depend upon its causing trouble; then no time should be lost in preventing infiltration or increase of the area of the disease. At this writing I have under treatment three cysts of extensive proportions, which are traceable directly to this cause. In the first case the swelling appeared in the nose, plugging the left nares, along with a perceptible enlargement between the internal canthus of the eye and the wing of the nose. Searching for the cause, I found the pulp in the first bicuspid dead and putrescent. This tooth was extracted by the patient's dentist, who claimed he did not find it abscessed, and the tooth, being of good quality, was cleansed, filled and replanted. The swelling somewhat diminished, but recurred in two years much increased in size. Examination of the alveolar process showed a normal condition. An opening was made into the tumor under the lip, in line with the wing of the nose, from which exuded a half ounce of thick greenish mucilaginous fluid. The sac had gradually increased until the substance of the superior maxillary bone had been destroyed from the point where I entered the sac to the nasal process, leaving only the periosteum intact. Even the inferior turbinated bone was destroyed. The walls of the nares offering the least resistance, the tumor gradually followed that direction until the left nares were completely plugged. The sac was curetted and the cavity treated and healed, care being observed to force back the periosteum so that the normal opening of the nares was restored.

The next case was where the pulp in the left central incisor had for many years been dead, presumably from a blow, as there was a slight fracture on the cutting edge. The pulp was opened into from the palatal surface of the tooth, and fully two drachms of pus flowed therefrom.

The pulp-canal was then cleansed and permanently filled, an incision made through the gums and periosteum from the labial surface, exposing the end of the root of the affected tooth. A deluge of thick, yellowish fluid flowed into the mouth—in all two ounces. It was found that all the bone extending from the right lateral to the left first bicuspid to the anterior boundary of the antrum and the nasal process was completely absorbed, leaving only the periosteum intact. There was also considerable bulging of the left nares and the roof of the mouth. This almost plugged the left nares; in fact, the floor of the nose was considerably elevated and the bone under it was completely destroyed. Fully two-thirds of the roots of the central incisors, left lateral cuspid and first bicuspid teeth penetrated this cavity. The patient never realized any trouble from this tumor, except occasionally for several years a little tenderness on pressure at the side of the nose, until about one month since, when the face suddenly swelled and was diagnosed by his dentist as the result of an alveolar abscess attached to the root in question. When the sac of the tumor was dissected away, it was found that immediately surrounding the affected tooth there was an independent sac, which was similar to that found in alveolar abscess and which contained the pus referred to.

The third, which was still more extensive, involved all the alveolar process of the left superior maxillary, from the third molar to the lateral incisor and extending to the molar. The face was considerably deformed by an enlargement below the molar about the size of a small hen's egg, which was apparently as firm as bone. Nearly the entire antrum was found occupied by this tumor, the contents being almost a jelly, of a slightly yellowish color, in quantity about three ounces. The etiology of the tumor I believe to be traceable to the back roots of the second molar, which had many years remained in position and were found penetrating the cyst.

These tumors are, fortunately, not numerous, and would be much less so if dead teeth were properly treated or promptly extracted. I could cite many cases of adenitis and inflammation of the ducts, also the bursa of the floor of the mouth, of osteoma and similar ailments, traceable to septic diseases of the pulp of the teeth, but I feel that it is not necessary to here dwell, or to impress you further on the importance of prompt and thorough removal of any such influence in the mouth.

In February, 1888, I operated on a case which illustrates clearly the lack of judgement of the dentist who filled a cavity over a septic pulp. The patient had the inferior left first molar filled with amalgam, from which the pulp had not been removed. The day following this filling the jaw around the tooth became sore and the face swollen. This condition continued to increase for several days. For two or more weeks she suffered much pain, when the swelling gradually diminished, leaving an indurated tumor near the apex of the roots, which continued to increase in firmness and constantly distressed the patient. At times the pain was quite severe. From the first of the trouble the patient was advised by the dentist to have nothing done with the tooth, as it was not at fault, while the physician and others advised its extraction. Two years later the patient consulted another dentist, who extracted the tooth, and found it in the condition as heretofore stated. This, however, did not check the growth of the tumor, which gradually increased in size until the face was considerably deformed. I was able to enucleate the tumor, and from its size could readily understand that the jaw-bone was nearly severed, there being not more than a quarter of an inch of the inferior border remaining, so completely had the growth of the tumor destroyed it. The tumor was osseous. The operation was done from within the mouth, thus avoiding the usual scarring, so disfiguring when the opening is made through the face. The case had been diagnosed a "sarcoma" and removal of half of the lower jaw was advised.—*Dominion Dental Journal.*

